# Three new species of the spider genus *Asceua* from Malaysia (Araneae, Zodariidae)

**DOI:** 10.3897/zookeys.789.24261

**Published:** 2018-10-10

**Authors:** Bao-Shi Zhang, Feng Zhang

**Affiliations:** 1 College of Life Sciences, Hebei Normal University, Shijiazhuang, Hebei 050024, P. R. China Hebei Normal University Hebei China; 2 Department of Biochemistry, Baoding University, Baoding, Hebei 071051, P. R. China Baoding University Baoding China; 3 The Key Laboratory of Invertebrate Systematics and Application, College of Life Sciences, Hebei University, Baoding, Hebei 071002, P. R. China Hebei University Baoding China

**Keywords:** description, distribution, Southeast Asia, taxonomy, Zodariid

## Abstract

Three new species of the genus *Asceua* Thorell, 1887, from the natural forests of Malaysia, are described as *Asceuabifurca***sp. n.** (♂♀), *A.curva***sp. n.** (♂), and *A.trimaculata***sp. n.** (♀). The genus *Asceua* is reported from Malaysia for the first time.

## Introduction

Members of the ant spider family Zodariidae Thorell, 1881 are small to medium-sized. It contains 85 genera and 1141 known species worldwide (World Spider Catalog 2018). Among them, 40 species are attributed to 5 genera (*Heliconilla* Dankittipakul, Jocqué & Singtripop, 2012, *Heradion* Dankittipakul & Jocqué, 2004, *Malayozodarion* Ono & Hashim, 2008, *Mallinella* Strand, 1906, and *Workmania* Dankittipakul, Jocqué & Singtripop, 2012) which have been reported from Malaysia. The genus *Asceua*was established by Thorell in 1887, with the type being *A.elegans* Thorell, 1887 from Myanmar. It was removed from synonymy with *Storena* Walckenaer, 1805 (Bosmans and van Hove 1986; Jocqué 1986). Jocqué (1991) later synonymized the genera *Suffucia* Simon, 1893 and *Doosia* Kishida, 1940 with *Asceua*. Members of this genus can be distinguished from other zodariids by their small size, laterally compressed bulb, developed cymbial fold, and the long and meandering copulatory ducts (Jocqué 1991).

At present, the genus includes 26 species worldwide (World Spider Catalog 2018). Among these, 22 are known from Southeast Asian countries that are close to Malaysia, including Japan, China (Southern part), Vietnam, Myanmar, Cambodia, Philippines and Indonesia; three are known from African countries and islands (Congo, Guinea-Bissau and the Comoros); and one is from Australia. Up until now, one described species is based on the specimen of unknown sex, eight are only known from female specimens and one only from male specimen. The species of this genus should be abundant, but are generally less well-known, and are worthy of further investigation in the future.

During the examination of spider collections from Malaysia, three new *Asceua* species were recognized and are described here as *Asceuabifurca* sp. n., *A.curva* sp. n., and *A.trimaculata* sp. n.

## Materials and methods

All specimens have been kept in 75% ethanol and were examined, drawn, and measured under a Tech XTL-II stereomicroscope equipped with an Abbe drawing device. Photos were taken with a Leica M205A stereomicroscope fitted with a Leica DFC550 Camera and LAS software (Ver. 4.6). Carapace length was measured medially from the anterior margin to the rear margin of the carapace. Eye sizes were measured as the maximum diameter of the lens in dorsal or frontal view. The measurements of legs are shown as total length (femur, patella, tibia, metatarsus, tarsus). Only one specimen of paratypes was measured. The epigynes were cleared in a warm solution of potassium hydroxide, and transferred to 75% ethanol for drawing. All measurements are in millimeters. All specimens studied are deposited in the Museum of Hebei University (**MHBU**), Baoding, China.

The following abbreviations are used:

**ALE** anterior lateral eyes;

**AME** anterior median eyes;

**C** conductor;

**CD** copulatory ducts;

**dRTA** dorsal apophysis of retrolateral tibial apophysis;

**E** embolus;

**MA** median apophysis;

**MOA** median ocular area;

**PLE** posterior lateral eyes;

**PME** posterior median eyes;

**RTA** retrolateral tibial apophysis;

**S** spermatheca;

**T** tegulum;

**vRTA** ventral apophysis of retrolateral tibial apophysis.

## Taxonomy

### Family Zodariidae Thorell, 1881

#### Genus *Asceua* Thorell, 1887 (Type species: *Asceuaelegans* Thorell, 1887)

##### Asceua bifurca sp. n.

Taxon classificationAnimaliaAraneaeZodariidae

http://zoobank.org/20CC40AA-232A-4157-B9A5-F7508F1EB525

Figs 1 , 2 , 3

###### Type material.

**Holotype** ♂, Malaysia, Sabah, Jalan Tambunan, Penampang, 05°48.739'N, 116°20.522'E, elev. 1583 m, 16 October 2015, Z.Z. Gao leg. **Paratypes**: 1 ♂ and 2 ♀, same data as holotype.

###### Diagnosis.

**Figure 1.**
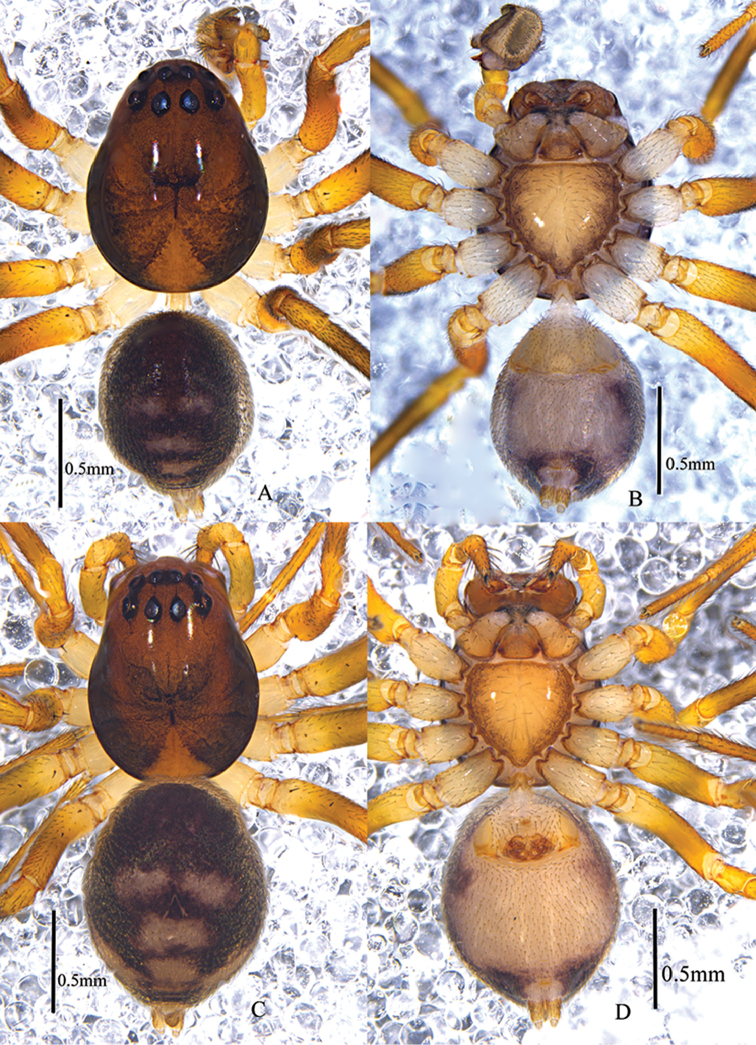
*Asceuabifurca* sp. n., male holotype (**A–B**) and female paratype (**C–D**) Habitus (**A, C** dorsal view **B, D** ventral view).

**Figure 2.**
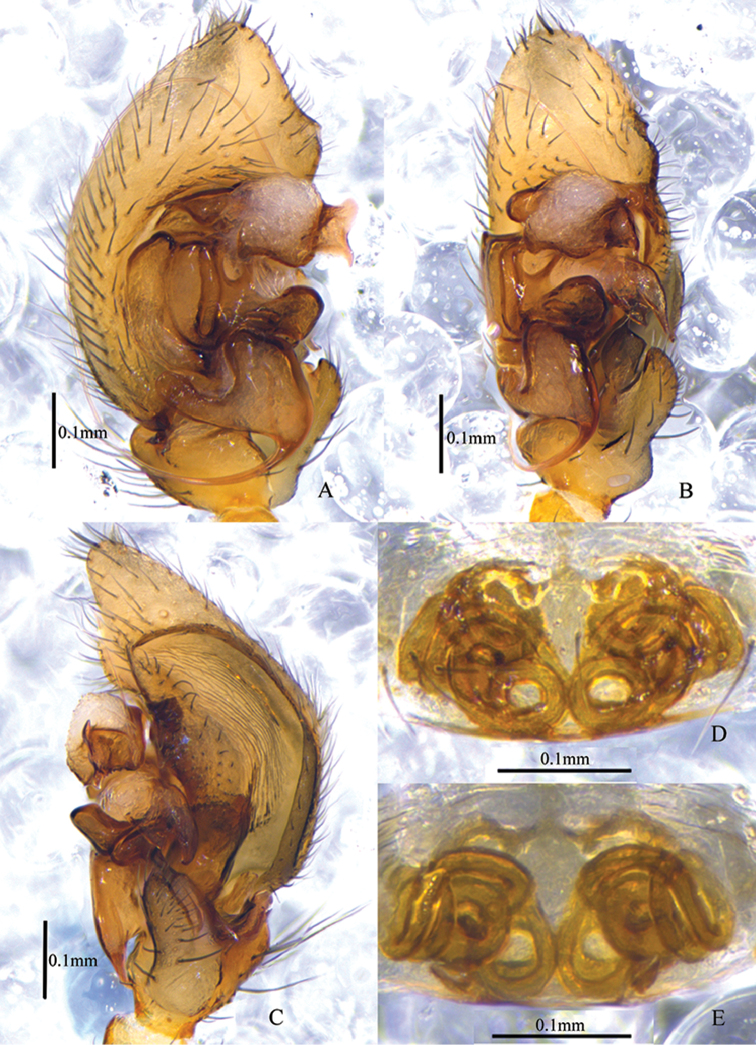
*Asceuabifurca* sp. n., male holotype (**A–C**) and female paratype (**D–E**) **A–C** Left male palp (**A** prolateral view **B** ventral view **C** retrolateral view) **D** Epigyne, ventral view **E** Epigyne, dorsal view.

**Figure 3.**
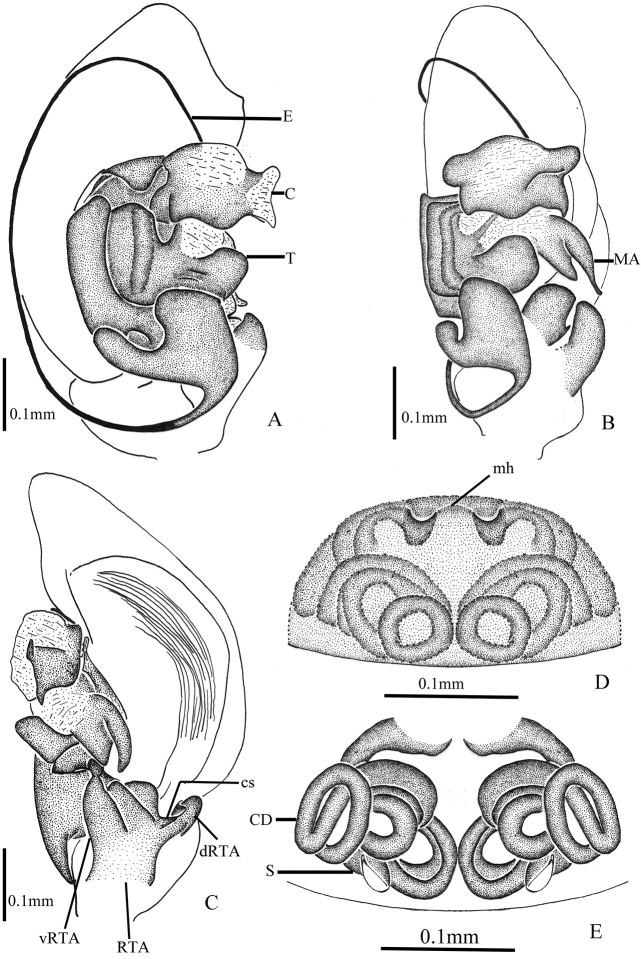
*Asceuabifurca* sp. n., male holotype (**A–C**) and female paratype (**D–E**) **A–C** Left male palp (**A** prolateral view **B** ventral view **C** retrolateral view) **D** Epigyne, ventral view **E** Epigyne, dorsal view. Abbreviations: cs, cuticularized sheet; mh, median hillock.

The males of *A.bifurca* are very similar to those of *A.radiosa* Jocqué, 1986 (from the Comoro Islands) in having a large concavity on the basal embolus and a short conductor. The two species can be easily distinguished by the conductor being bifurcated in the new species, while it is not bifurcated in *A.radiosa*. The posterior part of the dorsal abdomen has three white median bands in the new species that are absent in *A.radiosa* (Figs 1A, B, 2A–C, 3A–C). The females of this new species resemble those of *A.piperata* Ono, 2004 (from Vietnam) in having a hillock between the two copulatory openings, but the two spermathecae are spaced by copulatory ducts in the new species while they are adjacent in *A.piperata* (Figs 2D, E, 3D, E).

###### Etymology.

The specific name is taken from the Latin word *bifurca*, in reference to the bifurcated tip of the conductor; adjective.

###### Description.

Male total length 2.11–2.18. Holotype total length 2.18; carapace 1.13 long, 0.86 wide; opisthosoma 1.00 long, 0.74 wide. Habitus shown as in Fig. 1A–B. Carapace shiny, brown, lateral margins dark brown, tegument smooth, median part with a wide V-shaped black patch in front of black fovea, posterior middle bright. Radial grooves dark brown. Clypeus 0.16 high, brown. Eye sizes and inter-distances: AME 0.08, ALE 0.09, PME 0.09, PLE 0.09; AME–AME 0.04, AME–ALE 0.04, ALE–ALE 0.37, PME–PME 0.06, PME–PLE 0.08, PLE–PLE 0.47, ALE–PLE 0.02. MOA 0.22 long, frontal width 0.19, back width 0.24. Chelicerae brown, with 2 promarginal teeth and 1 retromarginal tooth, and terminal part armed with black hairs. Endites yellow brown, apices bright and furnished with dense black hairs. Labium triangular, 0.13 long, 0.12 wide, brown, median part with a semi-circular dark brown patch. Sternum 0.58 long, 0.50 wide, brown, lateral margin dark, median part bright and shiny, furnished with sparse black setae. Coxae of legs white, other sections brown, each femur with two dorsal spines, the distal part of tibia I bright. Measurements of legs: I 2.02 (0.40 + 0.20 + 0.61 + 0.38 + 0.43), II 1.84 (0.43 + 0.14 + 0.49 + 0.40 + 0.38), III 2.17 (0.62 + 0.21 + 0.51 + 0.46 + 0.37), IV 3.03 (0.61 + 0.33 + 0.72 + 0.95 + 0.42). Leg formula: 4312. Opisthosoma oval, covered with black short hairs, with a shiny and lanceolate dorsal scutum. Dorsum of opisthosoma black, with a pair of white median patches, followed by three transversal median bands, the first two bands wide and the third one narrow; anterior part of venter yellowish, posterior part white, and with a pair of lateral black patches, spinnerets brown, ringed with black.

Palp (Figs 2A–C, 3A–C). Coxae of palps white, other sections brown; length to width ratio of femur 2.6, length to width ratio of patella 1.2; RTA broad, ventral pointed apophysis broad and with trifurcate top, one of the forks longer than the other two forks, dorsal pointed apophysis thumb-like, with a cuticularized sheet situated between ventral and dorsal apophyses; cymbium with broad lateral fold which is wrinkly and with some hairs; conductor short, the tip bifurcated and sclerotized; distal median apophysis bifurcated; embolic base broad and almost an inverted triangle, with a large concavity on the apical margin.

Female total length 2.21–2.34. One of the paratypes total length 2.34; carapace 1.10 long, 0.84 wide; opisthosoma 1.25 long, 0.92 wide. Habitus as in Fig. 1C–D. Clypeus 0.15 high. Eye sizes and inter-distances: AME 0.07, ALE 0.09, PME 0.09, PLE 0.09; AME–AME 0.04, AME–ALE 0.03, ALE–ALE 0.37, PME–PME 0.05, PME–PLE 0.09, PLE–PLE 0.47, ALE–PLE 0.04. MOA 0.24 long, frontal width 0.17, back width 0.20. Labium 0.21 long, 0.24 wide. Sternum 0.53 long, 0.55 wide. Measurements of legs: I 2.07 (0.44 + 0.17+ 0.59 + 0.47 + 0.40), II 1.77 (0.44 + 0.11 + 0.41 + 0.47 + 0.34), III 1.99 (0.51 + 0.16 + 0.41 + 0.59 + 0.32), IV 2.53 (0.59 + 0.23 + 0.73 + 0.65 + 0.33). Leg formula: 4132. Dorsum of opisthosoma black, anterior median part dark brown and lack-lustre, followed by a pair of white patches and three transversal bands, lateral parts with a pair of white oblique patches. Color of ventral opisthosoma and spinnerets as in male.

Epigyne (Figs 2D, E, 3D, E). Plate of epigyne approx. 1.9 times wider than long, the posterior margins of copulatory openings and the anterior margin of median hillock W-shaped; spermathecae small and oval, almost as wide as the copulatory ducts, situated posteriorly and well-spaced (approx. 6 times the spermathecal diameter).

###### Distribution.

Malaysia (Sabah).

##### Asceua curva sp. n.

Taxon classificationAnimaliaAraneaeZodariidae

http://zoobank.org/280DE826-2A03-4081-87AC-EB17C530FCA2

Figs 4 , 5

###### Type material.

**Holotype** ♂, Malaysia, Sabah, Pitas, 06°29.598'N, 117°18.499'E, elev. 45 m, 20 October 2015, Z.Z. Gao leg.

###### Diagnosis.

**Figure 4.**
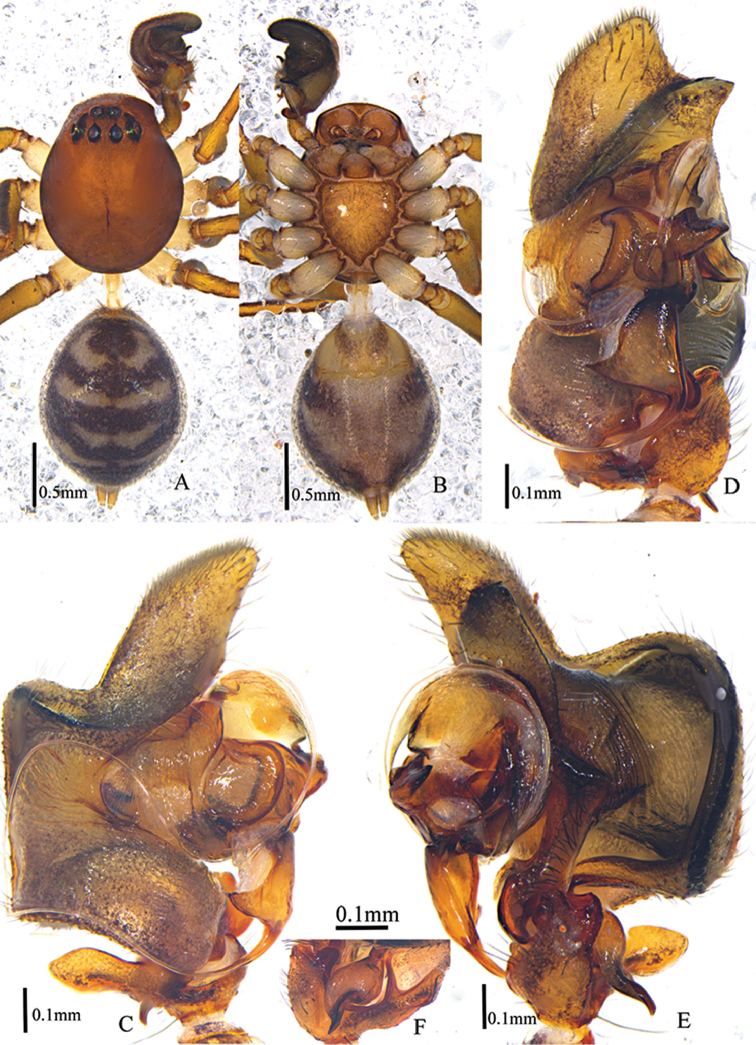
*Asceuacurva* sp. n., male holotype (**A–F**). **A–B** Habitus (**A** dorsal view **B** ventral view) **C–E** Left male palp (**C** prolateral view **D** ventral view **E** retrolateral view) **F** posterior projection of cymbium.

**Figure 5.**
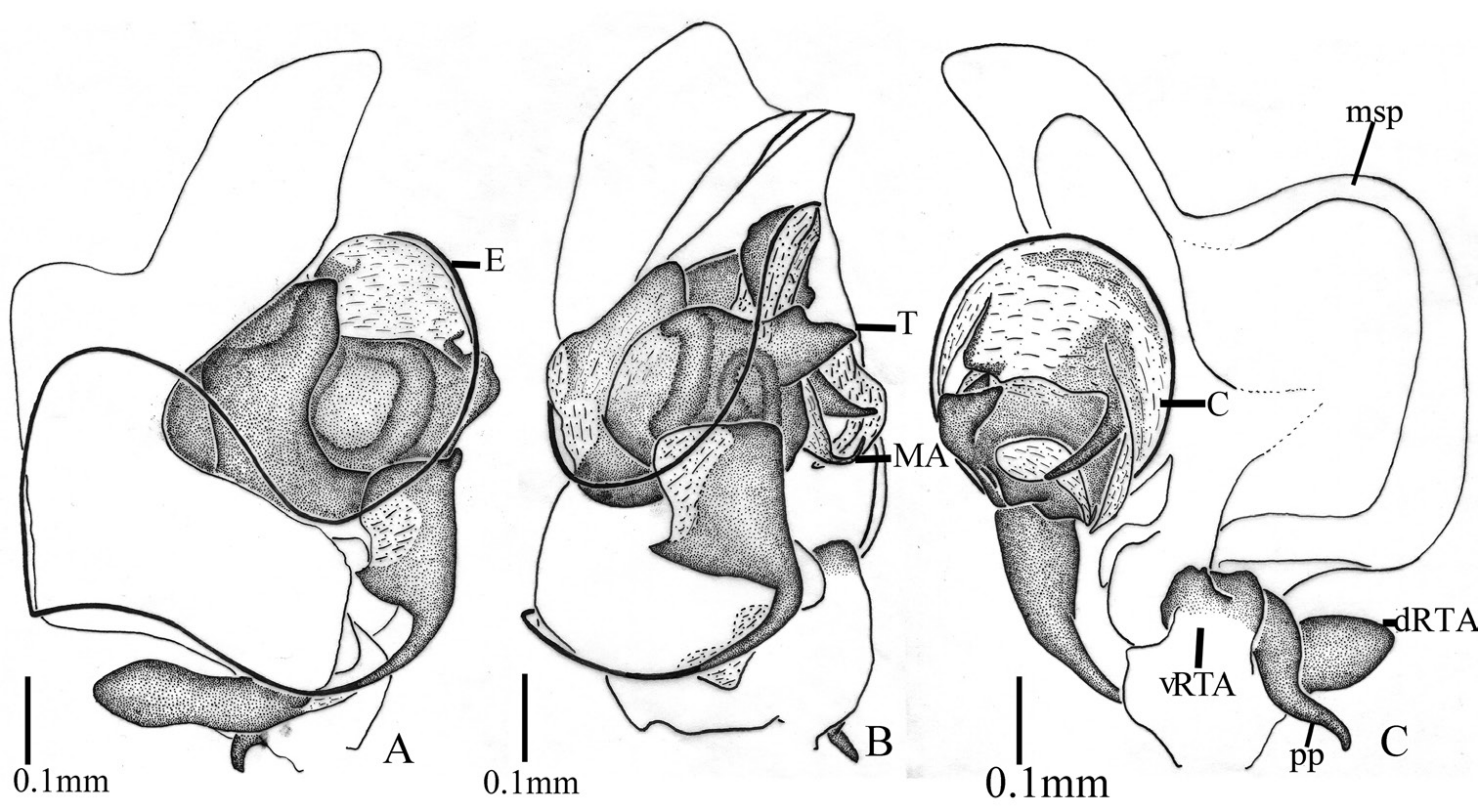
*Asceuacurva* sp. n. (**A–C**). Left palp of the male holotype (**A** prolateral view **B** ventral view **C** retrolateral view). Abbreviations: pp, posterior projection; msp, median semi-circular projection.

The male of this species resembles *A.wallacei* Bosmans & Hillyard, 1990 (from Sulawesi, Indonesia) in having the very complicated copulatory organ. The two species can be easily distinguished by: the thinner and longer retrolateral pointed processes of the distal conductor in the new species, which is shorter and bifurcated in *A.wallacei*; the longer posterior projection of the cymbium in the new species, which is shorter in *A.wallacei*; and the hook-like median apophysis which is almost straight in *A.wallacei* (Figs 4C–F, 5A–C).

###### Etymology.

The specific name is from the Latin word *curvus*, in reference to the shape of the posterior projection of the cymbium; adjective.

###### Description.

Male (holotype): Total length 3.15; carapace 1.39 long, 1.12 wide; opisthosoma 1.49 long, 1.17 wide. Habitus as in Fig. 4A–B. Carapace shiny, brown, lateral margins dark brown, part of carapace swollen, radial grooves inconspicuous. Clypeus 0.30 high, brown. Eye sizes and inter-distances: AME 0.11, ALE 0.08, PME0.08, PLE 0.13; AME–AME 0.05, AME–ALE 0.02, ALE–ALE 0.43, PME–PME 0.11, PME–PLE 0.10, PLE–PLE 0.59, ALE–PLE 0.02. MOA 0.27 long, frontal width 0.25, back width 0.27. Chelicerae brown, with two promarginal teeth and one retromarginal tooth, and terminal part armed with black hairs. Endites brown, apices bright and furnished with dense black hairs. Labium triangular, 0.20 long, 0.25 wide, brown, median part with a semicircular dark brown patch. Sternum 0.68 long, 0.66 wide, brown, lateral margin slightly dark brown, furnished with sparse black setae. Coxae of legs white, other sections brown, each femur with two dorsal spines, tibiae with long longitudinal dark stripes. Measurements of legs: I 3.74 (0.77 + 0.31 + 1.24 + 1.07 + 0.35), II 3.45 (0.89 + 0.36 + 0.94 + 0.88 + 0.38), III 3.46 (0.78 + 0.34 + 0.85 + 1.02 + 0.47), IV 3.80 (0.79 + 0.36 + 1.28 + 0.99 + 0.38). Leg formula: 4132. Opisthosoma covered with grey short hairs, dorsal scutum violin-like, dark brown. Dorsum of opisthosoma black, with a pair of white transversal chevrons, followed by three pairs of transversal stripes, the first two pairs being conjoint in the middle of the opisthosoma; anterior part of venter yellow, posterior part grey and lateral with two pairs of black oblique stripes, spinnerets brown.

Palp (Figs 4C–F, 5A–C). Tibia with two broad apophyses: dorsal apophysis and ventral apophysis, with a large concavity between them, in which fits a posterior projection of the cymbium; cymbium with a median semi-circular projection, which appears to be strongly excavated below in lateral view; tip of median apophysis hook-like; conductor large and semi-circular, with retrolateral and posterior pointed processes, not very chitinised except for the retrolateral processes; embolar base triangular; thread-like embolus very long, at first running to dorsal cymbium, then turning to ventral palp and following dorsal margin of conductor.

Female unknown.

###### Distribution.

Malaysia (Sabah).

###### Remarks.

Eight described *Asceua* species from nearby countries are only based on female specimens: *A.amabilis* Thorell, 1897 (from Myanmar), *A.anding* Zhang, Zhang & Jia, 2012 (from China), *A.daoxian* Yin, 2012 (from China), *A.elegans* Thorell, 1887 (from Myanmar), *A.kunming* Song & Kim, 1997 (from China), *A.longji* Barrion et al. 2013 (from China), *A.piperata* Ono, 2004 (from Vietnam), and *A.quinquestrigata* (Simon, 1905) (from Java). The patterns of the dorsal opisthosoma of these species are different by comparisons of illustrations and descriptions. The first pair of transversal chevrons are reniform in the new species, but are oval or long ovoid in all the other species, except for *A.quinquestrigata*. However, the new species can be distinguished from *A.quinquestrigata* by the broad bands on its posterior opisthosoma, which are only small in *A.quinquestrigata*. Also, the other white patches and transversal stripes on the opisthosoma of the new species contrasts with the lack of stripes in the other seven species except for *A.piperata*. However, the new species can be distinguished from *A.piperata* by its immaculate carapace. This new species is thus less likely to be conspecific with any of these 8 species that are only known from female specimens.

##### Asceua trimaculata sp. n.

Taxon classificationAnimaliaAraneaeZodariidae

http://zoobank.org/3212AFBA-61D7-439B-99C9-83E096C934BD

Fig. 6

###### Type material.

**Holotype** ♀, Malaysia, Pahang, Cameron Highlands, Tanah Rata, 04°27.791'N, 101°22.091'E, elev. 1380 m, 22 October 2015, Z.Z. Gao leg. **Paratype**: 1 ♀, same data as holotype.

###### Diagnosis.

**Figure 6.**
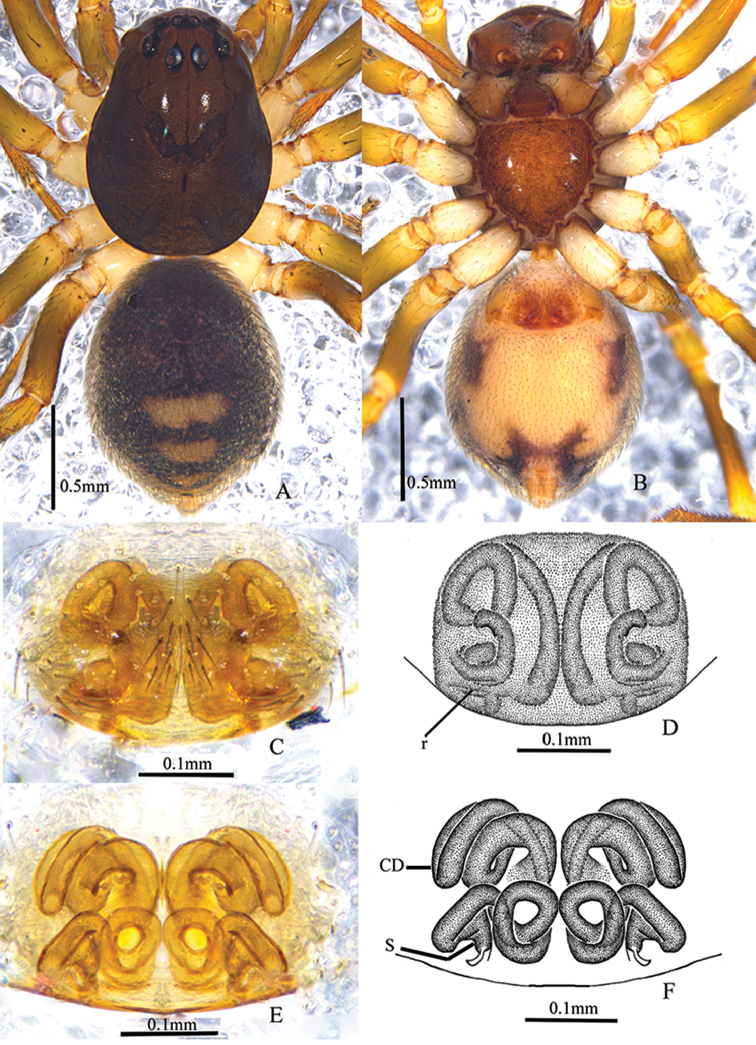
*Asceuatrimaculata* sp. n., female holotype (**A–F**) **A–B** Habitus (**A** dorsal view **B** ventral view) **C–F** Epigyne (**C, D** ventral view **E, F** dorsal view). Abbreviation: r, ridge.

The females of this new species resemble those of *A.lejeunei* Jocqué, 1991 (from Congo) in having widely spaced copulatory openings, but can be distinguished by the absence of the paired patches of dorsal opisthosoma which are present in *A.lejeunei* (Fig. 6A–F).

###### Etymology.

The specific name is from the Latin words *tri*- and *maculata*, in reference to the three patches on the dorsal opisthosoma.

###### Description.

Female total length 2.33–2.48. Holotype total length 2.48; carapace 1.21 long, 0.91 wide; opisthosoma 1.24 long, 0.95 wide. Habitus as in Figs 6A–B. Carapace, dark brown, median part with a black V-shaped patch and a longitudinal black thin band, radial grooves black. Clypeus 0.24 high, dark brown. Eye sizes and interdistances: AME 0.07, ALE 0.09, PME 0.08, PLE 0.09; AME–AME 0.02, AME–ALE 0.02, ALE–ALE 0.34, PME–PME 0.05, PME–PLE 0.12, PLE–PLE 0.46, ALE–PLE 0.05. MOA 0.26 long, frontal width 0.16, back width 0.21. Chelicerae dark brown, with two promarginal teeth and one retromarginal tooth, and terminal part armed with black hairs. Endites yellow brown, apices bright and furnished with dense black hairs. Labium triangular, 0.25 long, 0.28 wide, dark brown. Sternum 0.59 long, 0.61 wide, dark brown, median part shiny, furnished with sparse black setae. Coxae of legs yellowish, other sections brown. Measurements of legs: leg I 2.82 (0.84 + 0.29 + 0.67 + 0.59 + 0.43), II 2.35 (0.68 + 0.23 + 0.49 + 0.58 + 0.37), III 2.06 (0.65 + 0.16 + 0.34 + 0.52 + 0.39), IV 2.93 (0.89 + 0.16 + 0.50 + 1.03 + 0.35). Leg formula: 4123. Opisthosoma covered with black short hairs, lanceolate dorsal scutum dark brown and with blunt edge. Dorsum of opisthosoma black, with three transverse white bands; anterior part of venter yellow brown, posterior part yellowish and with a pair of lateral black patches, spinnerets brown and ringed with black.

Epigyne (Fig. 6C–F). Plate of epigyne approx. 1.3 times wider than long, copulatory openings situated almost at the middle part of epigyne, posterior epigynum with a pair of ridges; long and winding copulatory ducts visible through integument; spermathecae small, situated posteriorly and well-spaced (approx. 8 times the spermathecal diameter).

Male unknown.

###### Distribution.

Malaysia (Pahang).

###### Remarks.

*Asceuaseptemmaculata* (Simon, 1893a) was described based only on a male specimen from Cambodia. The patterning of the dorsal opisthosoma differ, in that the pairs of white patches present in *A.septemmaculata* are absent in the new species, and it is unlikely that the latter is conspecific with *A.septemmaculata*.

###### Comments.

There are five *Asceua* species in the adjacent region that are lacking illustrations: *A.bimaculata* (Simon, 1904) (from Vietnam), *A.heliophila* (Simon, 1893b) (from Philippines), *A.septemmaculata*, *A.amabilis* and *A.quinquestrigata*. The descriptions of the sexual organs were very simple. The three new species described here have to be distinguished by different patterns of the dorsal opisthosoma. *Asceuatrimaculata* sp. n. lacks pairs of white patches that all the five known species above possess. *Asceuabifurca* sp. n. differs from the five species by the rectangular white bands on its dorsal opisthosoma. *Asceuacurva* sp. n. differs from them by possessing the chevron patterning.

## Supplementary Material

XML Treatment for Asceua bifurca

XML Treatment for Asceua curva

XML Treatment for Asceua trimaculata

## References

[B1] Barrion AT Barrion-Dupo ALA Catindig JLA Villareal SC Cai D Yuan QH Heong KL (2013) New species of spiders (Araneae) from Hainan Island, China. UPLB Museum Publications in Natural History 3: 1–103.

[B2] Bosmans R Hillyard P (1990) Spiders of the family Zodariidae from Sulawesi, Indonesia (Arachnida: Araneae: Zodariidae). Bulletin of the British Arachnological Society 8: 147–160.

[B3] Bosmans R van Hove M (1986) A revision of the afrotropical representatives of the genus Langbiana Hogg (Araneae: Zodariidae). Bulletin of the British Arachnological Society 7: 17–28.

[B4] Jocqué R (1986) Ant-eating spiders from the Comoros (Araneae, Zodariidae). Revue de Zoologie Africaine 100: 307–312.

[B5] Jocqué R (1991) A generic revision of the spider family Zodariidae (Araneae). Bulletin of the American Museum of Natural History 201: 1–160.

[B6] Ono H (2004) Spiders of the family Zodariidae (Araneae) from Dambri, Lam Dong Province, southern Vietnam. Bulletin of the National Science Museum, Tokyo (A), 30: 67–75.

[B7] Simon E (1893a) Histoire naturelle das araignées. Paris 1: 257–488.

[B8] Simon E (1893b) Arachnides. In: Voyage de M E. Simon aux îles Philippines (Mars et Avril 1890). 6e Mémoire.Annales de la Société Entomologique de France 62: 65–80.

[B9] Simon E (1904) Arachnides recueillis par M. A. Pavie en Indochine – Mission Pavie en Indochine 1879–1895. III. Recherches sur l’histoire naturelles de l’Indochine Orientale. Paris, 270–295.

[B10] Simon E (1905) Arachnides de Java, recueillis par le Prof. K. Kraepelin en 1904. Mitteilungen aus dem Naturhistorischen Museum in Hamburg 22: 49–73.

[B11] Song DX Kim JP (1997) On seven new species of the family Zodariidae (Araneae) from China. Korean Arachnologica 13(1): 7–17.

[B12] Thorell T (1887) Viaggio di L. Fea in Birmania e regioni vicine. II. Primo saggio sui ragni birmani. Annali del Museo Civico di Storia Naturale di Genova 25: 5–417.

[B13] Thorell T (1897) Viaggio di Leonardo Fea in Birmania e regioni vicine. LXXIII. Secondo saggio sui Ragni birmani. I. Parallelodontes. Tubitelariae. Annali del Museo Civico di Storia Naturale di Genova (2)17[37]: 161–267.

[B14] World Spider Catalog (2018) World Spider Catalog. Natural History Museum Bern. http://wsc.nmbe.ch [version 19.0; Accessed 17 January 2018]

[B15] Yin CM Peng XJ Yan HM Bao YH Xu X Tang G Zhou QS Liu P (2012) Fauna Hunan: Araneae in Hunan, China. Hunan Science and Technology Press, Changsha, 1590 pp.

[B16] Zhang BS Zhang F Jia XM (2012) Two new species of the ant spider genus Asceua Thorell, 1887 (Araneae: Zodariidae) from China. Zootaxa 3307: 62–68.

